# Large Scale Meta-Analyses of Fasting Plasma Glucose Raising Variants in *GCK*, *GCKR*, *MTNR1B* and *G6PC2* and Their Impacts on Type 2 Diabetes Mellitus Risk

**DOI:** 10.1371/journal.pone.0067665

**Published:** 2013-06-28

**Authors:** Haoran Wang, Lei Liu, Jinzhao Zhao, Guanglin Cui, Chen Chen, Hu Ding, Dao Wen Wang

**Affiliations:** 1 Department of Internal Medicine, Tongji Hospital, Tongji Medical College, Huazhong University of Science and Technology, Wuhan, China; 2 Genetic Diagnosis Center, Tongji Hospital, Tongji Medical College, Huazhong University of Science and Technology, Wuhan, China; Gentofte University Hospital, Denmark

## Abstract

**Background:**

The evidence that the variants *GCK* rs1799884, *GCKR* rs780094, *MTNR1B* rs10830963 and *G6PC2* rs560887, which are related to fasting plasma glucose levels, increase the risk of type 2 diabetes mellitus (T2DM) is contradictory. We therefore performed a meta-analysis to derive a more precise estimation of the association between these polymorphisms and T2DM.

**Methods:**

All the publications examining the associations of these variants with risk of T2DM were retrieved from the MEDLINE and EMBASE databases. Using the data from the retrieved articles, we computed summary estimates of the associations of the four variants with T2DM risk. We also examined the studies for heterogeneity, as well as for bias of the publications.

**Results:**

A total of 113,025 T2DM patients and 199,997 controls from 38 articles were included in the meta-analysis. Overall, the pooled results indicated that *GCK* (rs1799884), *GCKR* (rs780094) and *MTNR1B* (rs10830963) were significantly associated with T2DM susceptibility (OR, 1.04; 95%CI, 1.01–1.08; OR, 1.08; 95%CI, 1.05–1.12 and OR, 1.05; 95%CI, 1.02–1.08, respectively). After stratification by ethnicity, significant associations for the *GCK*, *MTNR1B* and *G6PC2* variants were detected only in Caucasians (OR, 1.09; 95%CI, 1.02–1.16; OR, 1.10; 95%CI, 1.08–1.13 and OR, 0.97; 95%CI, 0.95–0.99, respectively), but not in Asians (OR, 1.02, 95% CI 0.98–1.05; OR, 1.01; 95%CI, 0.98–1.04 and OR, 1.12; 95%CI, 0.91–1.32, respectively).

**Conclusions:**

Our meta-analyses demonstrated that *GCKR* rs780094 variant confers high cross-ethnicity risk for the development of T2DM, while significant associations between *GCK*, *MTNR1B* and *G6PC2* variants and T2DM risk are limited to Caucasians.

## Introduction

Previous epidemiological studies have provided compelling evidence that fasting plasma glucose (FPG) levels that are on the high side of the normoglycemic range are associated with increased risk of type 2 diabetes mellitus (T2DM) [1], [2]. Recently, multiple genome wide association studies (GWASs) performed in populations of European descent have identified common sequence variants in the promoter region of glucokinase (*GCK*, rs1799884), glucokinase regulator protein (*GCKR*, rs780094), islet specific glucose-6-phosphatase (*G6PC2*, rs560887) and melatonin receptor 1B (*MTNR1B*, rs10830963) to be the variants that most influence FPG levels [3]–[5], with an effect size of >0.029 mmol/l per risk allele. Moreover, the significant associations between these variants and FPG were well replicated in other populations, including Asians and Africans [6], [7].

*GCK* encodes the key enzyme for the first step of glycolysis and is expressed only in liver and pancreatic islet beta cells [8]. Its activity is subject to inhibition by a regulatory protein, *GCKR* [9]. *G6PC2* is also known as the encoding gene for islet-specific glucose-6-phosphatase catalytic subunit-related protein (*IGRP*), which is expressed in a highly pancreatic beta-cell-specific manner. But its catalytic activity has not been clearly described so far [10]. *MTNR1B* encodes a melatonin receptor that is found mainly in the brain. However, the presence of this receptor in islets suggests a possible association between its function and insulin secretion [11]. Given their biological relevance to glucose metabolism, it is no surprise that variants in these genes have been associated with FPG levels and T2DM.

Because of the significant impact of these variants on FPG, numerous studies have investigated further the association between these variants and T2DM risk. Rose *et al*. found the *GCK* rs1799884 polymorphism was associated with impaired glucose regulation [12]. Sparso *et al*. reported that the G-allele of *GCKR* rs780094 polymorphism was associated with a modest increased risk of T2DM [13]. In two large prospective studies, Lyssenko *et al*. provided evidence that the risk genotype of the *MTNR1B* rs10830963 variant could predict future T2DM [11]. Dupuis *et al*. reported a significant association between the *G6PC2* rs560887 variant and T2DM risk [3]. Furthermore, Reiling *et al*. demonstrated that there were combined effects of these four single nucleotide polymorphisms (SNPs) on FPG levels and T2DM risk [5]. However, in many other association studies, negative results were reported for these four SNPs, especially in studies performed in Asian populations. For example, Tam *et al*. failed to validate the association between genetic variants in *GCK*, *GCKR*, *MTNR1B*, *G6PC2* and T2DM in a Chinese population [14], and this was consistent with the result of a study by Rees *et al*. in a south Asian population [15]. Given the discrepancies between the results of these studies and the low power of some of the small-scale association studies to detect small effect size results, we performed a comprehensive meta-analysis to give a more precise estimate of the associations between genetic variations in these four genes and T2DM risk.

## Methods

### Search Strategy

We conducted a systematic literature search (up to December 2012) of the MEDLINE and EMBASE databases in accordance with the preferred reporting items for systematic reviews and meta-analysis (PRISMA) statement [16]. For the search terms, we used gene name (*GCK*, *GCKR*, *MTNR1B* and *G6PC2*) and disease name (type 2 diabetes mellitus, T2DM or diabetes) to retrieve the association studies between genetic variants in *GCK*, *GCKR*, *MTNR1B* and *G6PC2* and risk of T2DM. The computer-aided search was supplemented by including additional studies retrieved from the references and citations of the originally identified articles and from the PubMed option ‘Related Articles’.

### Selection

Although several SNPs in the four studied genes have previously been linked to FPG levels and T2DM, only those variants that were studied in a total of >50,000 cases were analyzed. As a result, four SNPs (namely rs1799884 in *GCK*, rs780094 in *GCKR*, rs10830963 in *MTNR1B* and rs560887 in *G6PC2*) were finally included. Studies that met all the following criteria were included: (1) published in English; (2) with primary outcomes of T2DM; (3) described ethnicity and numbers of the study population; (4) provided the odds ratio (OR) with 95% confidence intervals (CIs) or enough genotype distribution data to calculate the ORs and 95% CIs. The exclusion criteria included: (1) not an association study for T2DM; (2) case-only study; (3) studied other SNPs; (4) meta-analysis. For duplicate publications, the study with the smaller data set was excluded.

### Data Extraction

The characteristics extracted from each study included ethnicity, year of publication, study design, number and male percentages of cases/controls, estimated OR and 95% confidence interval, genotype distribution or allele frequency. Two authors (H.W. and L.L.) extracted data independently and in duplicate. All disagreements and uncertainties were discussed and resolved by consensus, with the involvement of another author (H.D.) if necessary.

### Study Quality Assessment

The same two authors assessed the quality of included studies independently according to a quality assessment scores which was developed based on traditional epidemiologic and genetic considerations [17], [18]. And total scores ranged from 0 (worst) to 12 (best). Details of the criteria that were used to develop the scoring system are available in the Table S1. Any discrepancies were adjudicated by another author (H.D.).

### Meta-analyses

Data analyses were performed as follows. Firstly, we calculated the pooled prevalence of each risk allele in various ethnic groups using the inverse variance method described previously [18]. Secondly, the influence of these variants on T2DM risk was assessed by pooling together the per-allele ORs weighted by their inverse variance from each independent study. And a random-effects model was used by default to summarize the data as it properly takes into account the inter-study heterogeneity [19]. Heterogeneity was qualitatively assessed using the Q test and quantitatively evaluated with the *I^2^* test. *I^2^* test values of 25%, 50% and 75% were considered low, moderate and high, respectively [20]. In the presence of significant heterogeneity (Q test, *p*<0.05), the source of heterogeneity was explored by fitting a co-variant (quality score, case sample size, mean age and gender distribution of cases and controls) in a meta-regression model. Furthermore, considering the possible impact of ethnic variations on the results, we divided the study populations into three ethnic subgroups, including Caucasians, Asians and others. And differences between the subgroups were compared using the χ^2^ based Q test [21]. Thirdly, to evaluate the reliability and stability of our results, publication bias was evaluated with Egger’s linear regression and Begger’s funnel plot [22], [23], and the influence of each study on the pooled-OR was investigated in a sensitivity test by excluding one study each time. All probability values were 2-sided, values of *p*<0.05 were considered to be statistically significant and values of *p*<10^−8^ were considered to have reached a genome-wide significance level. All analyses were performed using the STATA software version 10.0 (Stata Corporation, College Station, TX, USA).

## Results

### Literature Search Results

**Figure 1:**
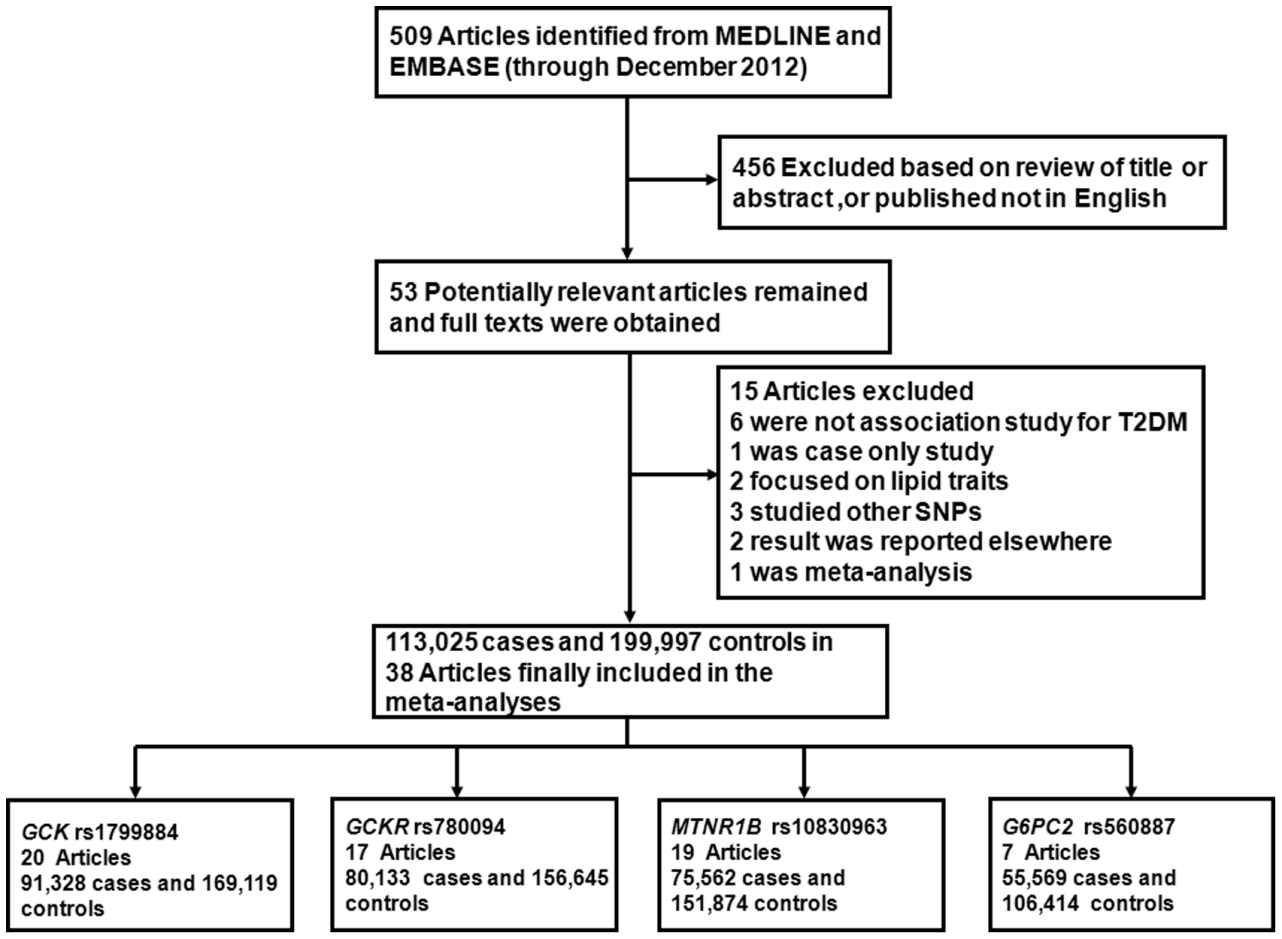
Flow diagram of study identification.

A total of 509 articles from MEDLINE and EMBASE were identified through the preliminary literature search up to December 2012. As shown in Figure 1, a total of 53 potentially relevant articles were retained on the basis of titles and abstracts, and full texts of these articles were obtained for detailed review. Fifteen articles were excluded for the following reasons: six were not association study for T2DM [24]–[29], one was case-only study [30], two focused on lipid traits [31], three were studies of other SNPs [32]–[34], two of the results were reported elsewhere [35], [36], and the remaining one was a meta-analysis [37]. Totally, 38 articles consisting of 113,025 cases and 199,997 controls were finally included [3]–[7], [11]–[15], [38]–[65]. Of all the studies included, 15 were studies of populations of European descent, 19 were studies of populations of Asian descent and 4 were studies of mixed/other ethnicities. The detailed characters of the included association studies are listed in Table 1.

**Table 1:** Characteristics of genetic association studies included in the current meta-analyses.

First author	Ethnicity			Year	Case			Control						*GCK*		*GCKR*	*G6PC2*	*MTNR1B*
					Number	Male%	Age	Number		Male%		Age		rs1799884		rs780094	rs560887	rs10830963
Rose et al. [12]	Danish			2005	1408	60.4	57	4441		46.54		45		√				
Sparso et al. [13]	Danish			2007	3878	59.4	61.8	4891		46.5		46.6				√		
Bouatia-Naji et al. [44]	French			2008	2972	62.2	50.4	4073		47.1		46.8					√	
Cauchi et al. [45]	French+Swiss			2008	2825	53.7	56.6	4472		37.3		45.6		√				
Holmkvist et al. [50]	Finnish			2008	132	50.8	51.7	2293		45.8		44.9		√				
Holmkvist et al. [50]	Swedish			2008	1872	78.3	NA	13666		63.7		45.5		√				
Vaxillaire et al. [64]	French			2008	2215	NA	NA	2251		50		47.7		√				
Ezzidi et al. [47]	Tunisian			2009	884	45.9	59.4	513		50.3		60		√				
Lyssenko et al. [11]	Swedish			2009	2063	64.9	45.5	13998		64.9		45.5						√
Lyssenko et al. [11]	Finnish			2009	138	50.8	44.9	2632		NA		NA						√
Qi et al. [59]	Chinese			2009	424	44.3	58.6	1908		44.3		58.8		√		√		
Reiling et al. [5]	Netherlands			2009	2628	55	64	2041		46		53		√			√	√
Ronn et al. [60]	Chinese			2009	1165	39.1	60.3	1105		31.6		59.4						√
Rose et al. [61]	Danish			2009	1408	60.4	57	4773		46.6		46.2					√	
Sparso et al. [62]	Danish			2009	1948	61.6	60.2	4905		46.4		46.2						√
Sparso et al. [62]	French			2009	183	48.9	47	2894		48.9		47						√
Sparso et al. [62]	French			2009	2622	NA	NA	4343		NA		NA						√
Bi et al. [43]		White American	2010		992	47	54.3		9937		47		54.3			√		
Bi et al. [43]		Black American	2010		772	38.3	53.5		3188		38.3		53.5			√		
Dupuis et al. [3]		Mixed	2010		40655	NA	NA		87022		NA		NA	√		√	√	√
Hu et al. [4]		Chinese	2010		3410	54.9	60.3		3412		40		50.1	√		√		√
Mohas et al. [55]		Hungarian	2010		321	53.6	61.3		172		28.5		56.5			√		
Onuma et al. [58]		Japanese	2010		506	55.3	60		402		53.2		59	√		√		
Takeuchi et al. [7]		Japanese	2010		5629	NA	NA		6406		NA		NA				√	
Takeuchi et al. [7]		Sri Lankan	2010		599	NA	NA		515		NA		NA				√	
Tam et al. [14]		Chinese	2010		1342	40.5	44.5		1644		45.4		24.6	√		√		√
Wen et al. [65]		Chinese	2010		1165	39.1	60.3		1136		31.1		59.1			√		
Been et al. [42]		Asian Indian	2011		1201	52.3	53.9		1021		52.4		50.7					√
Cho et al. [40]		East Asian	2011		6952	NA	NA		11865		NA		NA	√		√		√
Dietrich et al. [46]		German	2011		103	NA	48		547		NA		48					√
Kooner et al. [41]		South Asian	2011		5561	NA	NA		14512		NA		NA	√		√		
Ling et al. [52]		Chinese		2011	1118	44.6	60.2		1161		42.7		56.5		√			
Ling et al. [53]		Chinese		2011	1118	44.6	60.2		1161		42.7		56.5					√
Ohshige et al. [57]		Japanese		2011	2839	60.5	62.8		2125		47.6		51.6	√				√
Olsson et al. [39]		Norwegian		2011	1322	48.9	68.4		1447		50.2		65.2					√
Rees et al. [15]		South Asian		2011	821	52.4	54.6		1167		52.9		56.3	√	√		√	√
Rees et al. [15]		South Asian		2011	857	45.3	56.9		417		52		54.9	√	√		√	√
Tabara et al. [38]		Japanese		2011	506	55.3	60		402		53.2		59					√
Cauchi et al. [6]		Moroccan		2012	1193	34.3	54		1055		30.3		58	√				
Cauchi et al. [6]		Tunisian		2012	1446	44.3	61		942		45.8		61	√				
Florez et al. [48]		American		2012	633	32.3	50.6		2890		32.3		50.6		√			√
Fujita et al. [49]		Japanese		2012	2632	NA	64.1		2050		NA		69.7	√				√
Iwata et al. [51]		Japanese		2012	1182	59.6	65.3		859		44.4		69.5	√	√			
Liu et al. [54]		Chinese		2012	424	44.3	58.6		2786		44.3		58.6					√
Ng et al. [56]		African American		2012	2806	38.1	47.3		4265		39.4		51.1	√	√			
Tabassum et al. [63]		Asian Indian		2012	5482	42	50.1		4588		43.9		48.2	√	√			
Tabassum et al. [63]		Indo-European		2012	1256	57.8	45		1209		56.6		50	√	√		?	?

NA: not available; √ represents this SNP was studied.

### Heterogeneous Association of the *GCK* rs1799884 Polymorphism with T2DM Risk

**Figure 2:**
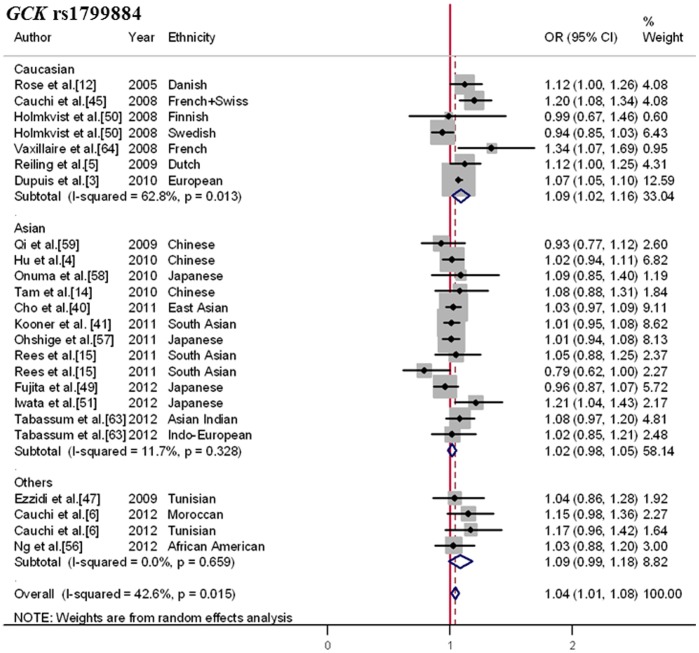
Forest plot for the association between *GCK* rs1799884 and T2DM. Pooled OR for the additive genetic model was shown under a random-effects model. Square sizes were proportional to weight of each study in the meta-analysis. Significant association was detected in Caucasians but not in Asians and others.

Not all researchers used the same SNPs. The most widely used was rs1799884. The remaining 5 articles used 2 additional SNPs, rs4607517 and rs730497. Based on 1000 genome project, the SNP rs1799884 was in strong linkage disequilibrium (LD) with rs4607517 (r^2^ = 1.0) and rs730497 (r^2^ = 1.0) across different racial populations (CEU, CHB, YRI), respectively. Therefore, the SNP rs1799884, which tags rs4607517 and rs730497, is probably the best proxy to evaluate the effect of this gene. Totally, 20 articles involving 91,328 cases and 169,119 controls were included to evaluate the effect of rs1799884 (or as proxy) for T2DM risk. As shown in Table S2, the pooled frequency of the minor A-allele was identical among Asians and Caucasians (minor allele frequency (MAF) = 0.16), while lower in others (MAF = 0.12). In the overall estimate (Figure 2), the minor A-allele of *GCK* rs1799884 was significantly associated with increased risk of diabetes (OR, 1.04; 95%CI, 1.01–1.08; *p* = 0.006), with moderate heterogeneity (Q = 40.09; I^2^ = 42.6%; *p* = 0.015).

After being stratified for ethnicity, significant difference between ethnic groups was detected (subgroup difference χ^2^ = 8.79; *p* = 0.012). The results indicated that the minor A-allele might be associated with an augmented T2DM risk (OR, 1.09; 95%CI, 1.02–1.16; *p* = 0.015) in Caucasians. However, no clear evidence for such an association was observed in either Asians (OR, 1.02; 95%CI, 0.98–1.05; *p* = 0.329) or others (OR, 1.09; 95%CI, 0.99–1.18; *p* = 0.075).

### Homogeneous Association of the *GCKR* rs780094 Polymorphism with T2DM Risk

**Figure 3:**
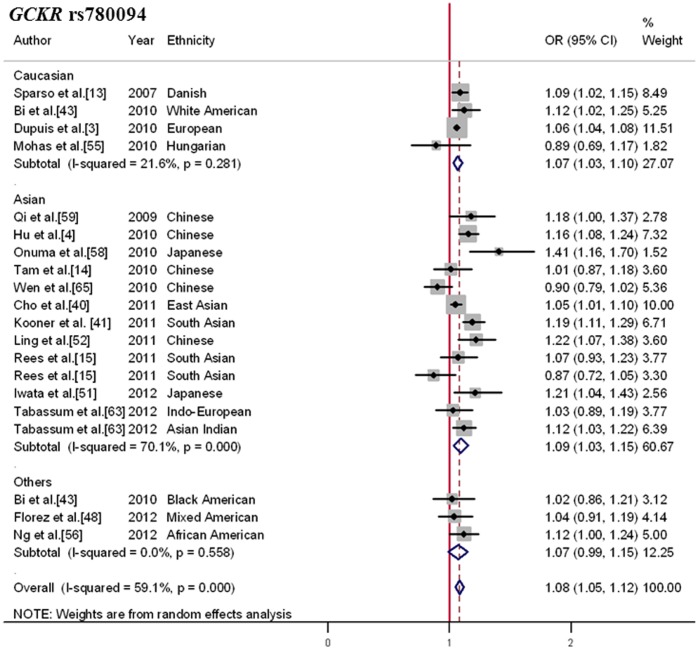
Forest plot for the association between *GCKR* rs780094 and T2DM. Pooled OR for the additive genetic model was shown under a random-effects model. Square sizes were proportional to weight of each study in the meta-analysis. Our meta-analyses demonstrated *GCKR* locus confers high cross-ethnicity risk for development of T2DM.

In total, 20 studies from 17 independent publications investigating the influence of the rs780094 on the risk of T2DM were combined, yielding a meta-analysis of data from 236,778 individuals (80,133 cases and 156,645 controls). As presented in Table S3, the pooled C-allele frequency was slightly lower in Caucasians (MAF = 0.62) than in Asians (MAF = 0.67), while much higher in African Americans (MAF = 0.82). In the overall estimate (Figure 3), a significant association was observed between the C-allele and elevated risk of T2DM (OR, 1.08; 95%CI, 1.05–1.12; *p* = 3.8×10^−6^) with high heterogeneity among studies (Q = 46.49; I^2^ = 59.1%; *p*<0.001). After being stratified for ethnicity, significant associations were observed both in Caucasians (OR, 1.07; 95%CI, 1.03–1.10; *p* = 1.3×10^−4^) and Asians (OR, 1.09; 95%CI, 1.03–1.15; *p* = 0.002), with no difference in ORs observed (subgroup difference χ^2^ = 1.34; *p* = 0.511).

### Heterogeneous Association of the *MTNR1B* rs10830963 Polymorphism with T2DM Risk

**Figure 4:**
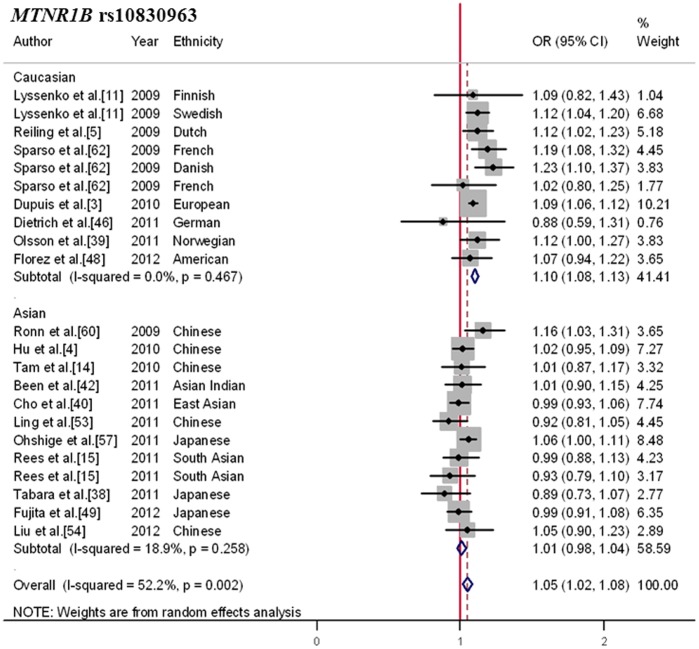
Forest plot for the association between *MTNR1B* rs10830963 and T2DM. Pooled OR for the additive genetic model was shown under a random-effects model. Square sizes were proportional to weight of each study in the meta-analysis. The result indicated that significant association was limited to Caucasians.

Meta-analysis on the relationship between rs10830963 and T2DM risk included 18 independent articles containing data from 227,436 subjects (75,562 cases and 151,874 controls). As shown in Table S4, the risk G-allele frequency was higher in Asians (MAF = 0.42) than in Caucasians (MAF = 0.30). In the overall estimate (Figure 4), the G-allele was significantly associated with increased risk of T2DM (OR, 1.05; 95%CI, 1.02–1.08; *p* = 0.002).

A high level of heterogeneity was observed between the included studies (Q = 43.96; I^2^ = 52.2%; *p* = 0.002), and an inconsistent effect was noted when studies were considered separately by ancestry (subgroup difference χ^2^ = 21.71; *p* = 3.2×10^−6^). Indeed, the association between the minor G-allele and T2DM risk was well replicated and reached a genome wide significance level in populations of Caucasians (OR, 1.10; 95%CI, 1.08–1.13; *p* = 6.7×10^−16^), but it is not replicable in Asians (OR, 1.01; 95%CI, 0.98–1.04; *p* = 0.547).

### Contrasting Effects of the *G6PC2* rs560887 Polymorphism on Risk of T2DM between Caucasians and Asians

**Figure 5:**
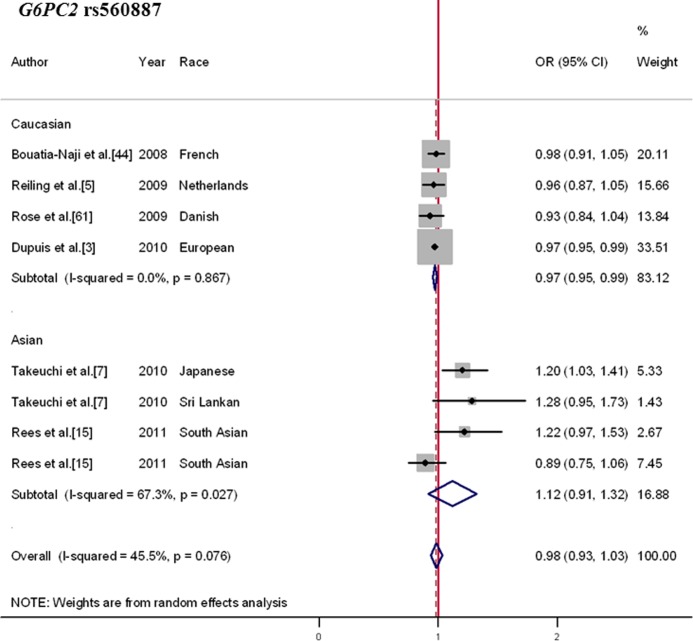
Forest plot for the association between *G6PC2* rs560887 and T2DM. Pooled OR for the additive genetic model was shown under a random-effects model. Square sizes were proportional to weight of each study in the meta-analysis. Contrasting results were detected between Caucasians and Asians.

We pooled data from 6 articles containing a total of 55,569 cases and 106,414 controls. As indicated in Table S5, the risk A-allele frequency was much lower in Asians (MAF = 0.04) than in Caucasians (MAF = 0.30). In the overall estimate (Figure 5), the association between the rs560887-G allele and T2DM risk was non-significant (OR, 0.98; 95%CI, 0.93–1.03; *p* = 0.458), with moderate heterogeneity (Q = 12.85; I^2^ = 45.5%; *p* = 0.002). However, when considered separately by ethnicity, a contrasting effect of this variant on T2DM was observed (subgroup difference χ^2^ = 2.94; *p* = 0.086). Results from Caucasian studies indicated the FPG-raising G-allele might be associated with a decreased risk of T2DM (OR, 0.97; 95%CI, 0.95–0.99; *p* = 0.001), with no heterogeneity observed (Q = 0.73; I^2^ = 0.0%; *p* = 0.867). Conversely, in Asians, the G-allele was associated with increased risk of T2DM, although statistically not significant (OR, 1.12; 95%CI, 0.91–1.32; *p* = 0.257). Given the low frequency and limited sample size of Asian studies, the current meta-analysis may be still be under-powered to provide conclusive insights into this issue.

### Meta-regression

In the meta-regression analyses, neither sample size, study quality, mean age of cases and controls nor sex distribution in cases and controls were significantly correlated with the magnitude of the genetic effect (all *p*>0.05).

### Publication Bias, Sensitivity Test

Based on Begger’s funnel plots (Figures S1–S4) and Egger’s linear regression, we didn’t detect any publication bias for all the pooled analyses (Egger’s test, all *p*>0.05). Besides, in the sensitivity test (Figures S5–S8), the leave-one-out influential analyses showed that no individual study would significantly modify the estimates, and this further confirmed the stability and reliability of the pooled results.

## Discussion

The present meta-analyses provided the most comprehensive evaluation of the associations between FPG-raising variants and T2DM risk. In the overall estimates comprising individuals from different ethnicities, significant associations with increased risk of T2DM were detected for the *GCK*, *GCKR* and *MTNR1B* variants, but not for the *G6PC2* variant. However, the results should be interpreted with caution when heterogeneity between Caucasians and Asians was detected. In particular, significant associations with T2DM risk were found in Caucasians for all four SNPs, whereas in Asians, no significant associations were detected for the *GCK*, *MTNR1B* and *G6PC2* variants.

Several possibilities may explain the divergence across diverse ethnic groups. First, the distributions of the SNPs were different between various ethnic populations. For instance, the allele A frequencies of rs560887 differ from 1.7% in Asians to 30.8% in Caucasians. Given that the low frequency of rs560887 in Asians, it may have limited statistical power to detect positive association with a small effect. Second, the genetic variant of interest might be in LD with other causal variants, and the extent of LD was reported to differ in some of study populations that were examined [66]. Third, there may be population-specific genetic effects as a result of gene-gene and gene-environment interactions [67], [68]. Asians have been reported to have unique risk factor profiles for developing diabetes that differ from those in Caucasians [69]. All the above-mentioned factors might have contributed to the heterogeneous association results across ethnic groups.

The power of genetic association studies is always limited by sample size especially when the effect of a genetic variant is small, as was the case for the above-mentioned variants. Combining data from many studies to form a large sample size allows small effects to be detected and more precise estimates to be obtained. This was the main strength of the current meta-analysis. However, there are several limitations that should be noted. First, most of the study subjects were of European ancestry, the Asian subgroup only contained about 15,000 cases. And further Asian studies are required to give more precise estimate of the genetic effects. Second, although an exhaustive literature search was done, some publications (especially those published not in English) and unpublished work would have been missed, and publication bias may potentially exist. Third, because no original individual data were available, we were not able to further investigate the cumulative effect of the included variants and the gene-environment interactions could not be investigated.

In conclusion, our meta-analysis has provided robust evidence that the *GCKR* rs780094 polymorphism is an important variant that confers high cross-ethnicity risk for development of T2DM. Conversely, significant associations between the *GCK*, *MTNR1B* and *G6PC2* variants and T2DM risk are limited to Caucasians, and the meta-analysis results of associations of those variants with T2DM are required for further evaluation in larger sample size in Asian population.

## Supporting Information

Figure S1 **Begg’s funnel plot of studies of the** ***GCK*** **rs1799884 variant and T2DM.** Each point represents a separate study for the indicated association. Egger’s test, t = −0.42, *p* = 0.678.
(TIF)

Figure S2 **Begg’s funnel plot of studies of the** ***GCKR*** **rs780094 variant and T2DM.** Each point represents a separate study for the indicated association. Egger’s test, t = 0.86, *p* = 0.401.
(TIF)

Figure S3 **Begg’s funnel plot of studies of the** ***MTNR1B*** **rs10830963 variant and T2DM.** Each point represents a separate study for the indicated association. Egger’s test, t = −1.31, *p* = 0.205.
(TIF)

Figure S4 **Begg’s funnel plot of studies of the** ***G6PC2*** **rs560887 variant and T2DM.** Each point represents a separate study for the indicated association. Egger’s test, t = 1.35, *p* = 0.225.
(TIF)

Figure S5 **Sensitivity analyses of the** ***GCK*** **rs1799884 variant in an additive model by omitting one study at a time.** The summary OR (95% CI) was indicated by each horizontal line when the labeled study was omitted and the reminders were reanalyzed.
(TIF)

Figure S6 **Sensitivity analyses of the** ***GCKR*** **rs780094 variant in an additive model by omitting one study at a time.** The summary OR (95% CI) was indicated by each horizontal line when the labeled study was omitted and the reminders were reanalyzed.
(TIF)

Figure S7 **Sensitivity analyses of the** ***MTNR1B*** **rs10830963 variant in an additive model by omitting one study at a time.** The summary OR (95% CI) was indicated by each horizontal line when the labeled study was omitted and the reminders were reanalyzed.
(TIF)

Figure S8 **Sensitivity analyses of the** ***G6PC2*** **variant in an additive model by omitting one study at a time.** The summary OR (95% CI) was indicated by each horizontal line when the labeled study was omitted and the reminders were reanalyzed.
(TIF)

Table S1 **Quality score assessment criteria.**
(DOCX)

Table S2 **Estimation of the pooled prevalence of the risk A-allele of** ***GCK*** **rs1799884.**
(DOCX)

Table S3 **Estimation of the pooled prevalence of the risk C-allele of** ***GCKR*** **rs780094.**
(DOCX)

Table S4 **Estimation of the pooled prevalence of the risk G-allele of** ***MTNR1B*** **rs10830963.**
(DOCX)

Table S5 **Estimation of the pooled prevalence of the risk A-allele of** ***G6PC2*** **rs560887.**
(DOCX)

Table S6 **PRISMA Checklist for the current meta-analysis.**
(DOC)

Table S7 **PRISMA Flow Diagram for the current meta-analysis.**
(DOC)

## References

[1] Piche ME, Arcand-Bosse JF, Despres JP, Perusse L, Lemieux S, et al (2004) What is a normal glucose value? Differences in indexes of plasma glucose homeostasis in subjects with normal fasting glucose. Diabetes Care 27: 2470–2477. 15451918 10.2337/diacare.27.10.2470

[2] Tirosh A, Shai I, Tekes-Manova D, Israeli E, Pereg D, et al (2005) Normal fasting plasma glucose levels and type 2 diabetes in young men. N Engl J Med 353: 1454–1462. 16207847 10.1056/NEJMoa050080

[3] Dupuis J, Langenberg C, Prokopenko I, Saxena R, Soranzo N, et al (2010) New genetic loci implicated in fasting glucose homeostasis and their impact on type 2 diabetes risk. Nat Genet 42: 105–116. 20081858 10.1038/ng.520 PMC3018764

[4] Hu C, Zhang R, Wang C, Yu W, Lu J, et al (2010) Effects of GCK, GCKR, G6PC2 and MTNR1B variants on glucose metabolism and insulin secretion. PLoS One 5: e11761. 20668700 10.1371/journal.pone.0011761 PMC2909258

[5] Reiling E, van ‘t Riet E, Groenewoud MJ, Welschen LM, van Hove EC, et al (2009) Combined effects of single-nucleotide polymorphisms in GCK, GCKR, G6PC2 and MTNR1B on fasting plasma glucose and type 2 diabetes risk. Diabetologia 52: 1866–1870. 19533084 10.1007/s00125-009-1413-9 PMC2723681

[6] Cauchi S, Ezzidi I, El Achhab Y, Mtiraoui N, Chaieb L, et al (2012) European genetic variants associated with type 2 diabetes in North African Arabs. Diabetes Metab 38: 316–323. 22463974 10.1016/j.diabet.2012.02.003

[7] Takeuchi F, Katsuya T, Chakrewarthy S, Yamamoto K, Fujioka A, et al (2010) Common variants at the GCK, GCKR, G6PC2-ABCB11 and MTNR1B loci are associated with fasting glucose in two Asian populations. Diabetologia 53: 299–308. 19937311 10.1007/s00125-009-1595-1

[8] Matsutani A, Janssen R, Donis-Keller H, Permutt MA (1992) A polymorphic (CA)n repeat element maps the human glucokinase gene (GCK) to chromosome 7p. Genomics 12: 319–325. 1740341 10.1016/0888-7543(92)90380-b

[9] Warner JP, Leek JP, Intody S, Markham AF, Bonthron DT (1995) Human glucokinase regulatory protein (GCKR): cDNA and genomic cloning, complete primary structure, and chromosomal localization. Mamm Genome 6: 532–536. 8589523 10.1007/BF00356171

[10] Martin CC, Bischof LJ, Bergman B, Hornbuckle LA, Hilliker C, et al (2001) Cloning and characterization of the human and rat islet-specific glucose-6-phosphatase catalytic subunit-related protein (IGRP) genes. J Biol Chem 276: 25197–25207. 11297555 10.1074/jbc.M101549200

[11] Lyssenko V, Nagorny CL, Erdos MR, Wierup N, Jonsson A, et al (2009) Common variant in MTNR1B associated with increased risk of type 2 diabetes and impaired early insulin secretion. Nat Genet 41: 82–88. 19060908 10.1038/ng.288 PMC3725650

[12] Rose CS, Ek J, Urhammer SA, Glumer C, Borch-Johnsen K, et al (2005) A −30G>A polymorphism of the beta-cell-specific glucokinase promoter associates with hyperglycemia in the general population of whites. Diabetes 54: 3026–3031. 16186409 10.2337/diabetes.54.10.3026

[13] Sparso T, Andersen G, Nielsen T, Burgdorf KS, Gjesing AP, et al (2008) The GCKR rs780094 polymorphism is associated with elevated fasting serum triacylglycerol, reduced fasting and OGTT-related insulinaemia, and reduced risk of type 2 diabetes. Diabetologia 51: 70–75. 18008060 10.1007/s00125-007-0865-z

[14] Tam CH, Ho JS, Wang Y, Lee HM, Lam VK, et al (2010) Common polymorphisms in MTNR1B, G6PC2 and GCK are associated with increased fasting plasma glucose and impaired beta-cell function in Chinese subjects. PLoS One 5: e11428. 20628598 10.1371/journal.pone.0011428 PMC2900202

[15] Rees SD, Hydrie MZ, O’Hare JP, Kumar S, Shera AS, et al (2011) Effects of 16 genetic variants on fasting glucose and type 2 diabetes in South Asians: ADCY5 and GLIS3 variants may predispose to type 2 diabetes. PLoS One 6: e24710. 21949744 10.1371/journal.pone.0024710 PMC3176767

[16] Liberati A, Altman DG, Tetzlaff J, Mulrow C, Gotzsche PC, et al (2009) The PRISMA statement for reporting systematic reviews and meta-analyses of studies that evaluate health care interventions: explanation and elaboration. PLoS Med 6: e1000100. 19621070 10.1371/journal.pmed.1000100 PMC2707010

[17] Attia J, Thakkinstian A, D’Este C (2003) Meta-analyses of molecular association studies: methodologic lessons for genetic epidemiology. J Clin Epidemiol 56: 297–303. 12767405 10.1016/s0895-4356(03)00011-8

[18] Thakkinstian A, McEvoy M, Minelli C, Gibson P, Hancox B, et al (2005) Systematic review and meta-analysis of the association between {beta}2-adrenoceptor polymorphisms and asthma: a HuGE review. Am J Epidemiol 162: 201–211. 15987731 10.1093/aje/kwi184

[19] Fleiss JL (1993) The statistical basis of meta-analysis. Stat Methods Med Res 2: 121–145. 8261254 10.1177/096228029300200202

[20] Higgins JP, Thompson SG, Deeks JJ, Altman DG (2003) Measuring inconsistency in meta-analyses. BMJ 327: 557–560. 12958120 10.1136/bmj.327.7414.557 PMC192859

[21] Petitti DB (2001) Approaches to heterogeneity in meta-analysis. Stat Med 20: 3625–3633. 11746342 10.1002/sim.1091

[22] Begg CB, Mazumdar M (1994) Operating characteristics of a rank correlation test for publication bias. Biometrics 50: 1088–1101. 7786990

[23] Egger M, Davey Smith G, Schneider M, Minder C (1997) Bias in meta-analysis detected by a simple, graphical test. BMJ 315: 629–634. 9310563 10.1136/bmj.315.7109.629 PMC2127453

[24] Brito EC, Lyssenko V, Renstrom F, Berglund G, Nilsson PM, et al (2009) Previously associated type 2 diabetes variants may interact with physical activity to modify the risk of impaired glucose regulation and type 2 diabetes: a study of 16,003 Swedish adults. Diabetes 58: 1411–1418. 19324937 10.2337/db08-1623 PMC2682680

[25] Chen G, Bentley A, Adeyemo A, Shriner D, Zhou J, et al (2012) Genome-wide association study identifies novel loci association with fasting insulin and insulin resistance in African Americans. Hum Mol Genet 21: 4530–4536. 22791750 10.1093/hmg/dds282 PMC3459464

[26] Ramos E, Chen G, Shriner D, Doumatey A, Gerry NP, et al (2011) Replication of genome-wide association studies (GWAS) loci for fasting plasma glucose in African-Americans. Diabetologia 54: 783–788. 21188353 10.1007/s00125-010-2002-7 PMC3052446

[27] Renstrom F, Shungin D, Johansson I, Investigators M, Florez JC, et al (2011) Genetic predisposition to long-term nondiabetic deteriorations in glucose homeostasis: Ten-year follow-up of the GLACIER study. Diabetes 60: 345–354. 20870969 10.2337/db10-0933 PMC3012192

[28] Saxena R, Hivert MF, Langenberg C, Tanaka T, Pankow JS, et al (2010) Genetic variation in GIPR influences the glucose and insulin responses to an oral glucose challenge. Nat Genet 42: 142–148. 20081857 10.1038/ng.521 PMC2922003

[29] Wagner R, Dudziak K, Herzberg-Schafer SA, Machicao F, Stefan N, et al (2011) Glucose-raising genetic variants in MADD and ADCY5 impair conversion of proinsulin to insulin. PLoS One 6: e23639. 21887289 10.1371/journal.pone.0023639 PMC3161735

[30] Bonetti S, Trombetta M, Boselli ML, Turrini F, Malerba G, et al (2011) Variants of GCKR affect both beta-cell and kidney function in patients with newly diagnosed type 2 diabetes: the Verona newly diagnosed type 2 diabetes study 2. Diabetes Care 34: 1205–1210. 21411509 10.2337/dc10-2218 PMC3114499

[31] Hishida A, Morita E, Naito M, Okada R, Wakai K, et al (2012) Associations of apolipoprotein A5 (APOA5), glucokinase (GCK) and glucokinase regulatory protein (GCKR) polymorphisms and lifestyle factors with the risk of dyslipidemia and dysglycemia in Japanese - a cross-sectional data from the J-MICC Study. Endocr J 59: 589–599. 22517333 10.1507/endocrj.ej11-0310

[32] Chambers JC, Zhang W, Zabaneh D, Sehmi J, Jain P, et al (2009) Common genetic variation near melatonin receptor MTNR1B contributes to raised plasma glucose and increased risk of type 2 diabetes among Indian Asians and European Caucasians. Diabetes 58: 2703–2708. 19651812 10.2337/db08-1805 PMC2768158

[33] Stancakova A, Paananen J, Soininen P, Kangas AJ, Bonnycastle LL, et al (2011) Effects of 34 risk loci for type 2 diabetes or hyperglycemia on lipoprotein subclasses and their composition in 6,580 nondiabetic Finnish men. Diabetes 60: 1608–1616. 21421807 10.2337/db10-1655 PMC3292337

[34] Vaxillaire M, Cavalcanti-Proenca C, Dechaume A, Tichet J, Marre M, et al (2008) The common P446L polymorphism in GCKR inversely modulates fasting glucose and triglyceride levels and reduces type 2 diabetes risk in the DESIR prospective general French population. Diabetes 57: 2253–2257. 18556336 10.2337/db07-1807 PMC2494697

[35] Prokopenko I, Langenberg C, Florez JC, Saxena R, Soranzo N, et al (2009) Variants in MTNR1B influence fasting glucose levels. Nat Genet 41: 77–81. 19060907 10.1038/ng.290 PMC2682768

[36] Balkau B, Lange C, Fezeu L, Tichet J, de Lauzon-Guillain B, et al (2008) Predicting diabetes: clinical, biological, and genetic approaches: data from the Epidemiological Study on the Insulin Resistance Syndrome (DESIR). Diabetes Care 31: 2056–2061. 18689695 10.2337/dc08-0368 PMC2551654

[37] Xia Q, Chen ZX, Wang YC, Ma YS, Zhang F, et al (2012) Association between the melatonin receptor 1B gene polymorphism on the risk of type 2 diabetes, impaired glucose regulation: a meta-analysis. PLoS One 7: e50107. 23226241 10.1371/journal.pone.0050107 PMC3511448

[38] Tabara Y, Osawa H, Kawamoto R, Onuma H, Shimizu I, et al (2011) Genotype risk score of common susceptible variants for prediction of type 2 diabetes mellitus in Japanese: the Shimanami Health Promoting Program (J-SHIPP study). Development of type 2 diabetes mellitus and genotype risk score. Metabolism 60: 1634–1640. 21550079 10.1016/j.metabol.2011.03.014

[39] Olsson L, Pettersen E, Ahlbom A, Carlsson S, Midthjell K, et al (2011) No effect by the common gene variant rs10830963 of the melatonin receptor 1B on the association between sleep disturbances and type 2 diabetes: results from the Nord-Trondelag Health Study. Diabetologia 54: 1375–1378. 21380592 10.1007/s00125-011-2106-8

[40] Cho YS, Chen CH, Hu C, Long J, Ong RT, et al (2012) Meta-analysis of genome-wide association studies identifies eight new loci for type 2 diabetes in east Asians. Nat Genet 44: 67–72. 10.1038/ng.1019 PMC3582398 22158537

[41] Kooner JS, Saleheen D, Sim X, Sehmi J, Zhang W, et al (2011) Genome-wide association study in individuals of South Asian ancestry identifies six new type 2 diabetes susceptibility loci. Nat Genet 43: 984–989. 21874001 10.1038/ng.921 PMC3773920

[42] Been LF, Hatfield JL, Shankar A, Aston CE, Ralhan S, et al (2012) A low frequency variant within the GWAS locus of MTNR1B affects fasting glucose concentrations: genetic risk is modulated by obesity. Nutr Metab Cardiovasc Dis 22: 944–951. 21558052 10.1016/j.numecd.2011.01.006 PMC3155734

[43] Bi M, Kao WH, Boerwinkle E, Hoogeveen RC, Rasmussen-Torvik LJ, et al (2010) Association of rs780094 in GCKR with metabolic traits and incident diabetes and cardiovascular disease: the ARIC Study. PLoS One 5: e11690. 20661421 10.1371/journal.pone.0011690 PMC2908550

[44] Bouatia-Naji N, Rocheleau G, Van Lommel L, Lemaire K, Schuit F, et al (2008) A polymorphism within the G6PC2 gene is associated with fasting plasma glucose levels. Science 320: 1085–1088. 18451265 10.1126/science.1156849

[45] Cauchi S, Nead KT, Choquet H, Horber F, Potoczna N, et al (2008) The genetic susceptibility to type 2 diabetes may be modulated by obesity status: implications for association studies. BMC Med Genet 9: 45. 18498634 10.1186/1471-2350-9-45 PMC2412856

[46] Dietrich K, Birkmeier S, Schleinitz D, Breitfeld J, Enigk B, et al (2011) Association and evolutionary studies of the melatonin receptor 1B gene (MTNR1B) in the self-contained population of Sorbs from Germany. Diabet Med 28: 1373–1380. 21711391 10.1111/j.1464-5491.2011.03374.x

[47] Ezzidi I, Mtiraoui N, Cauchi S, Vaillant E, Dechaume A, et al (2009) Contribution of type 2 diabetes associated loci in the Arabic population from Tunisia: a case-control study. BMC Med Genet 10: 33. 19368707 10.1186/1471-2350-10-33 PMC2678106

[48] Florez JC, Jablonski KA, McAteer JB, Franks PW, Mason CC, et al (2012) Effects of genetic variants previously associated with fasting glucose and insulin in the Diabetes Prevention Program. PLoS One 7: e44424. 22984506 10.1371/journal.pone.0044424 PMC3439414

[49] Fujita H, Hara K, Shojima N, Horikoshi M, Iwata M, et al (2012) Variations with modest effects have an important role in the genetic background of type 2 diabetes and diabetes-related traits. J Hum Genet 57: 776–779. 22992776 10.1038/jhg.2012.110

[50] Holmkvist J, Almgren P, Lyssenko V, Lindgren CM, Eriksson KF, et al (2008) Common variants in maturity-onset diabetes of the young genes and future risk of type 2 diabetes. Diabetes 57: 1738–1744. 18332101 10.2337/db06-1464

[51] Iwata M, Maeda S, Kamura Y, Takano A, Kato H, et al (2012) Genetic risk score constructed using 14 susceptibility alleles for type 2 diabetes is associated with the early onset of diabetes and may predict the future requirement of insulin injections among Japanese individuals. Diabetes Care 35: 1763–1770. 22688542 10.2337/dc11-2006 PMC3402252

[52] Ling Y, Li X, Gu Q, Chen H, Lu D, et al (2011) Associations of common polymorphisms in GCKR with type 2 diabetes and related traits in a Han Chinese population: a case-control study. BMC Med Genet 12: 66. 21569451 10.1186/1471-2350-12-66 PMC3112072

[53] Ling Y, Li X, Gu Q, Chen H, Lu D, et al (2011) A common polymorphism rs3781637 in MTNR1B is associated with type 2 diabetes and lipids levels in Han Chinese individuals. Cardiovasc Diabetol 10: 27. 21470412 10.1186/1475-2840-10-27 PMC3079619

[54] Liu C, Wu Y, Li H, Qi Q, Langenberg C, et al (2010) MTNR1B rs10830963 is associated with fasting plasma glucose, HbA1C and impaired beta-cell function in Chinese Hans from Shanghai. BMC Med Genet 11: 59. 20398260 10.1186/1471-2350-11-59 PMC2873324

[55] Mohas M, Kisfali P, Jaromi L, Maasz A, Feher E, et al (2010) GCKR gene functional variants in type 2 diabetes and metabolic syndrome: do the rare variants associate with increased carotid intima-media thickness? Cardiovasc Diabetol 9: 79. 21114848 10.1186/1475-2840-9-79 PMC3009616

[56] Ng MC, Saxena R, Li J, Palmer ND, Dimitrov L, et al (2013) Transferability and fine mapping of type 2 diabetes Loci in african americans: the candidate gene association resource plus study. Diabetes 62: 965–976. 23193183 10.2337/db12-0266 PMC3581206

[57] Ohshige T, Iwata M, Omori S, Tanaka Y, Hirose H, et al (2011) Association of new loci identified in European genome-wide association studies with susceptibility to type 2 diabetes in the Japanese. PLoS One 6: e26911. 22046406 10.1371/journal.pone.0026911 PMC3202571

[58] Onuma H, Tabara Y, Kawamoto R, Shimizu I, Kawamura R, et al (2010) The GCKR rs780094 polymorphism is associated with susceptibility of type 2 diabetes, reduced fasting plasma glucose levels, increased triglycerides levels and lower HOMA-IR in Japanese population. J Hum Genet 55: 600–604. 20574426 10.1038/jhg.2010.75

[59] Qi Q, Wu Y, Li H, Loos RJ, Hu FB, et al (2009) Association of GCKR rs780094, alone or in combination with GCK rs1799884, with type 2 diabetes and related traits in a Han Chinese population. Diabetologia 52: 834–843. 19241058 10.1007/s00125-009-1290-2

[60] Ronn T, Wen J, Yang Z, Lu B, Du Y, et al (2009) A common variant in MTNR1B, encoding melatonin receptor 1B, is associated with type 2 diabetes and fasting plasma glucose in Han Chinese individuals. Diabetologia 52: 830–833. 19241057 10.1007/s00125-009-1297-8

[61] Rose CS, Grarup N, Krarup NT, Poulsen P, Wegner L, et al (2009) A variant in the G6PC2/ABCB11 locus is associated with increased fasting plasma glucose, increased basal hepatic glucose production and increased insulin release after oral and intravenous glucose loads. Diabetologia 52: 2122–2129. 19669124 10.1007/s00125-009-1463-z

[62] Sparso T, Bonnefond A, Andersson E, Bouatia-Naji N, Holmkvist J, et al (2009) G-allele of intronic rs10830963 in MTNR1B confers increased risk of impaired fasting glycemia and type 2 diabetes through an impaired glucose-stimulated insulin release: studies involving 19,605 Europeans. Diabetes 58: 1450–1456. 19324940 10.2337/db08-1660 PMC2682679

[63] Tabassum R, Chauhan G, Dwivedi OP, Mahajan A, Jaiswal A, et al (2013) Genome-wide association study for type 2 diabetes in indians identifies a new susceptibility locus at 2q21. Diabetes 62: 977–986. 23209189 10.2337/db12-0406 PMC3581193

[64] Vaxillaire M, Veslot J, Dina C, Proenca C, Cauchi S, et al (2008) Impact of common type 2 diabetes risk polymorphisms in the DESIR prospective study. Diabetes 57: 244–254. 17977958 10.2337/db07-0615

[65] Wen J, Ronn T, Olsson A, Yang Z, Lu B, et al (2010) Investigation of type 2 diabetes risk alleles support CDKN2A/B, CDKAL1, and TCF7L2 as susceptibility genes in a Han Chinese cohort. PLoS One 5: e9153. 20161779 10.1371/journal.pone.0009153 PMC2818850

[66] Teo YY, Ong RT, Sim X, Tai ES, Chia KS (2010) Identifying candidate causal variants via trans-population fine-mapping. Genet Epidemiol 34: 653–664. 20839287 10.1002/gepi.20522

[67] Hunter DJ (2005) Gene-environment interactions in human diseases. Nat Rev Genet 6: 287–298. 15803198 10.1038/nrg1578

[68] Yang Q, Khoury MJ, Sun F, Flanders WD (1999) Case-only design to measure gene-gene interaction. Epidemiology 10: 167–170. 10069253

[69] Weber MB, Oza-Frank R, Staimez LR, Ali MK, Narayan KM (2012) Type 2 diabetes in Asians: prevalence, risk factors, and effectiveness of behavioral intervention at individual and population levels. Annu Rev Nutr 32: 417–439. 22524185 10.1146/annurev-nutr-071811-150630

