# Supplementary figures and images for: Large Scale Meta-Analyses of Fasting Plasma Glucose Raising Variants in GCK, GCKR, MTNR1B and G6PC2 and Their Impacts on Type 2 Diabetes Mellitus Risk
Source: PLoS One. 2013 Jun 28;8(6):e67665. doi: 10.1371/journal.pone.0067665 (PMC3695948; doi:10.1371/journal.pone.0067665)

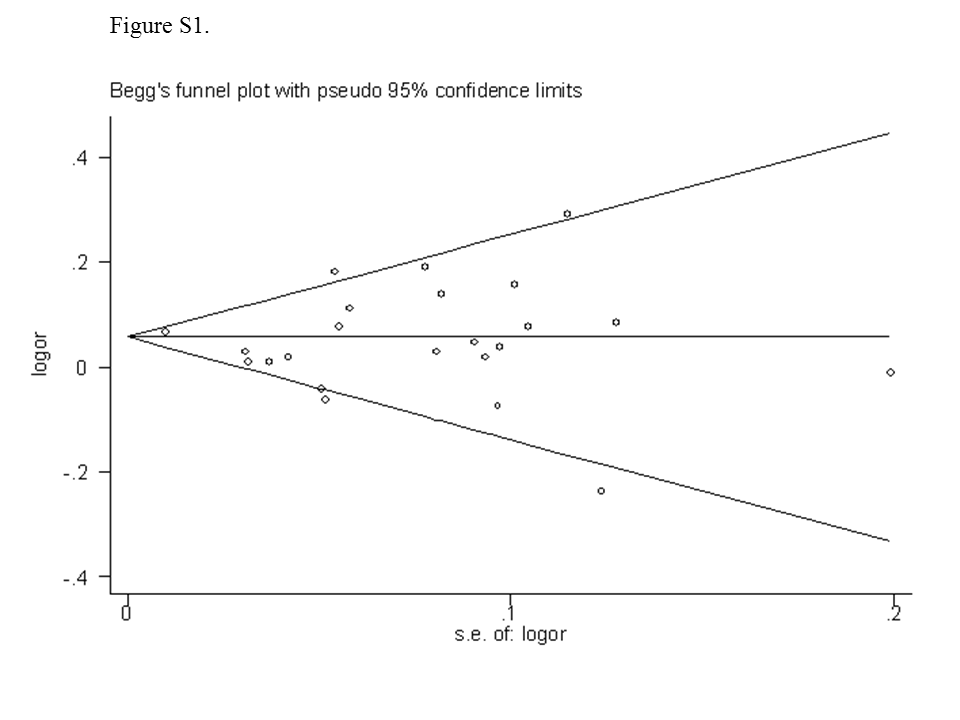

Supplement: Figure S1 — Begg’s funnel plot of studies of the GCK rs1799884 variant and T2DM. Each point represents a separate study for the indicated association. Egger’s test, t = −0.42, p = 0.678. (TIF) [file pone.0067665.s001.tif]

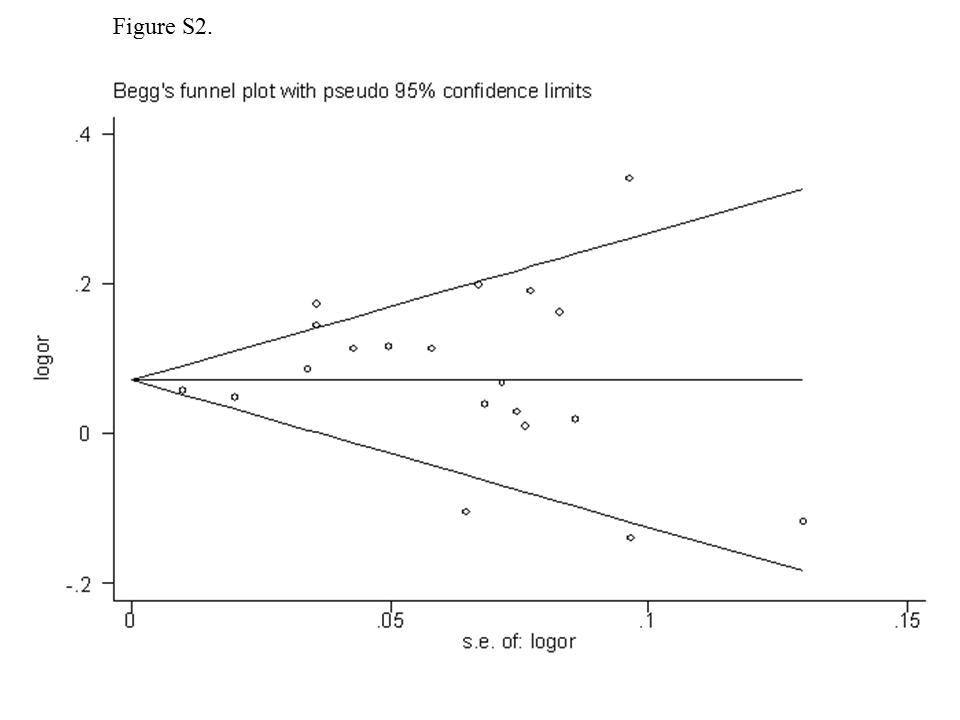

Supplement: Figure S2 — Begg’s funnel plot of studies of the GCKR rs780094 variant and T2DM. Each point represents a separate study for the indicated association. Egger’s test, t = 0.86, p = 0.401. (TIF) [file pone.0067665.s002.tif]

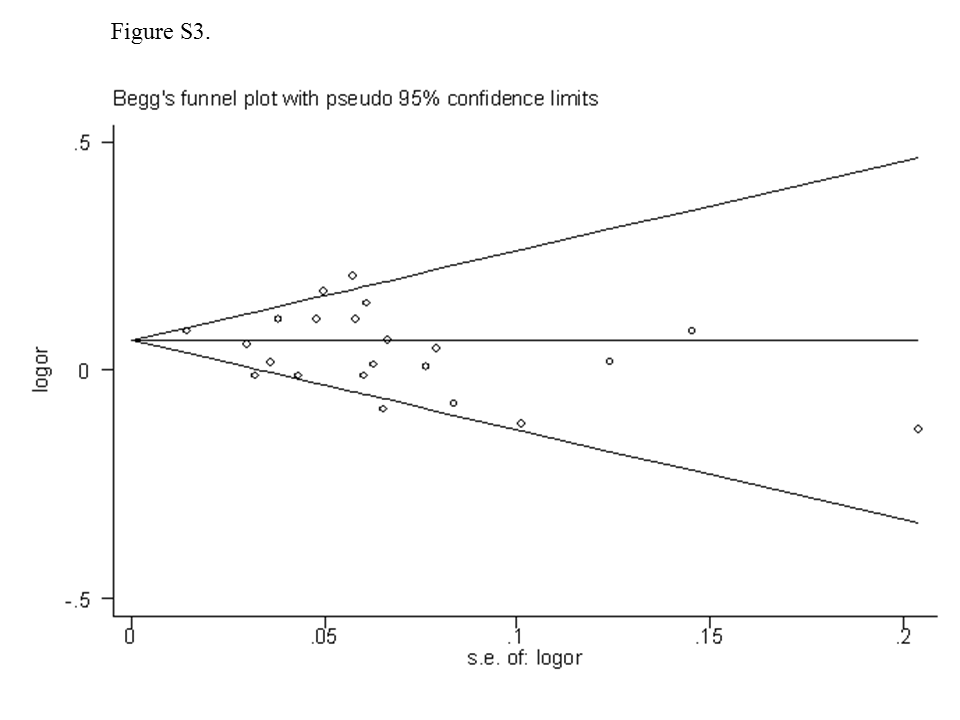

Supplement: Figure S3 — Begg’s funnel plot of studies of the MTNR1B rs10830963 variant and T2DM. Each point represents a separate study for the indicated association. Egger’s test, t = −1.31, p = 0.205. (TIF) [file pone.0067665.s003.tif]

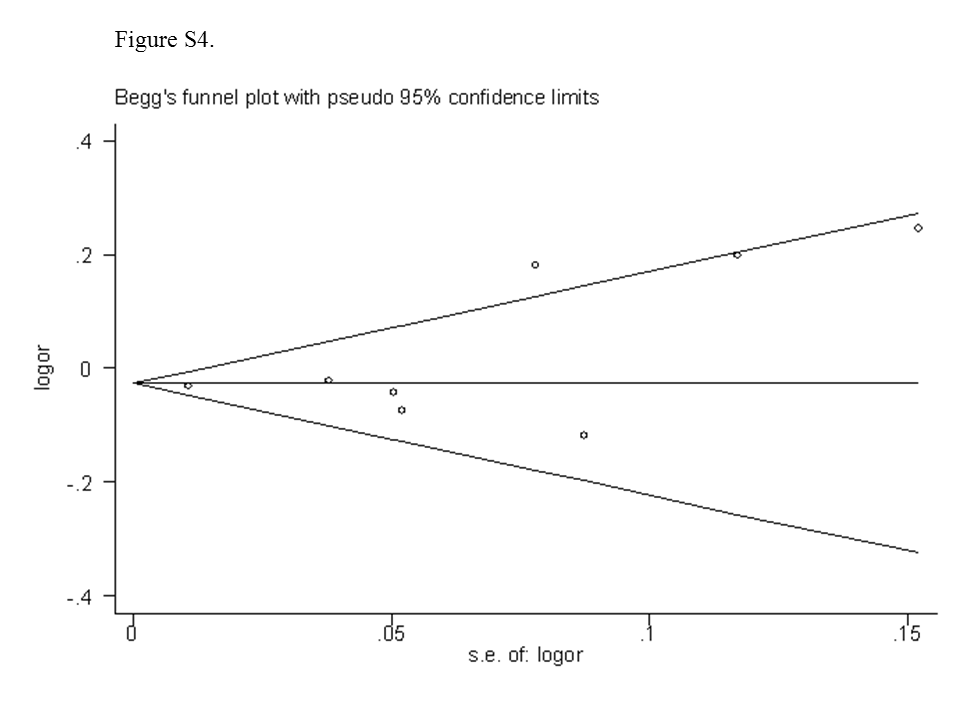

Supplement: Figure S4 — Begg’s funnel plot of studies of the G6PC2 rs560887 variant and T2DM. Each point represents a separate study for the indicated association. Egger’s test, t = 1.35, p = 0.225. (TIF) [file pone.0067665.s004.tif]

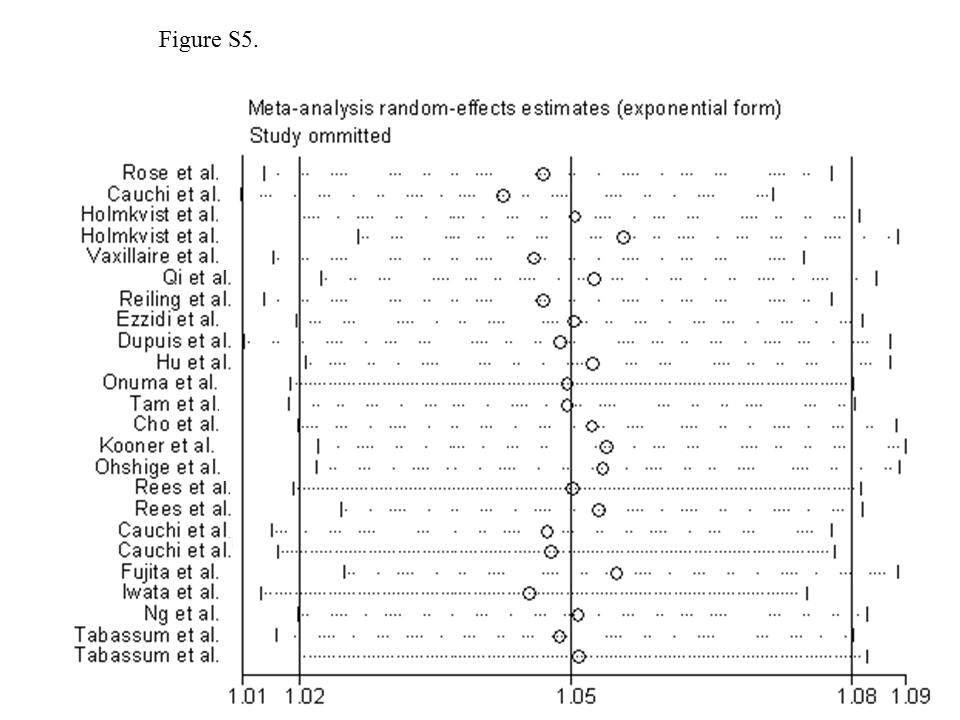

Supplement: Figure S5 — Sensitivity analyses of the GCK rs1799884 variant in an additive model by omitting one study at a time. The summary OR (95% CI) was indicated by each horizontal line when the labeled study was omitted and the reminders were reanalyzed. (TIF) [file pone.0067665.s005.tif]

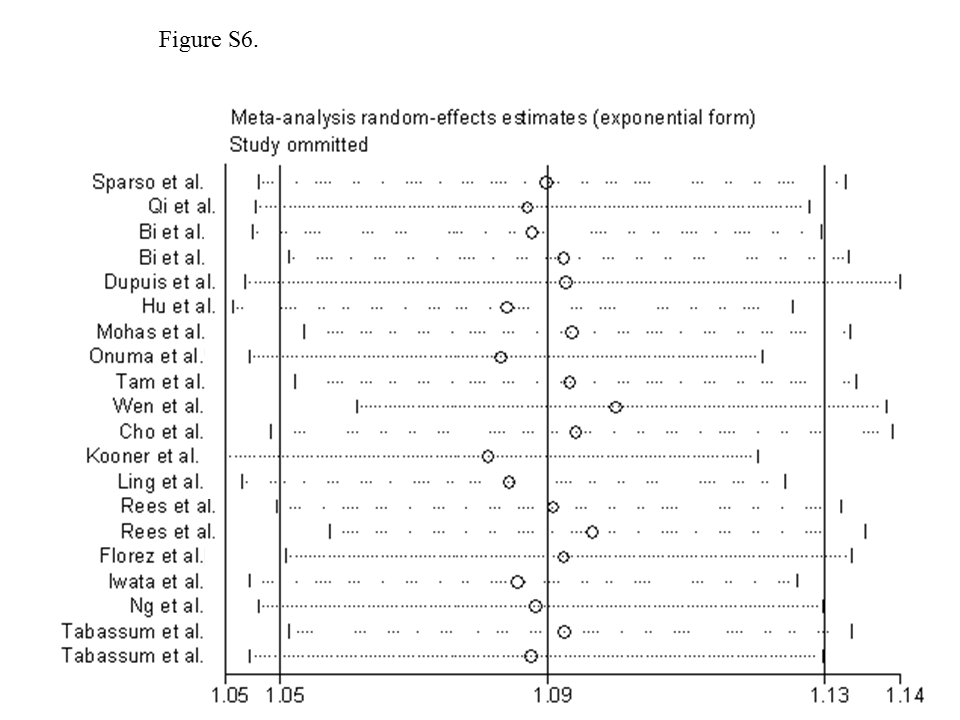

Supplement: Figure S6 — Sensitivity analyses of the GCKR rs780094 variant in an additive model by omitting one study at a time. The summary OR (95% CI) was indicated by each horizontal line when the labeled study was omitted and the reminders were reanalyzed. (TIF) [file pone.0067665.s006.tif]

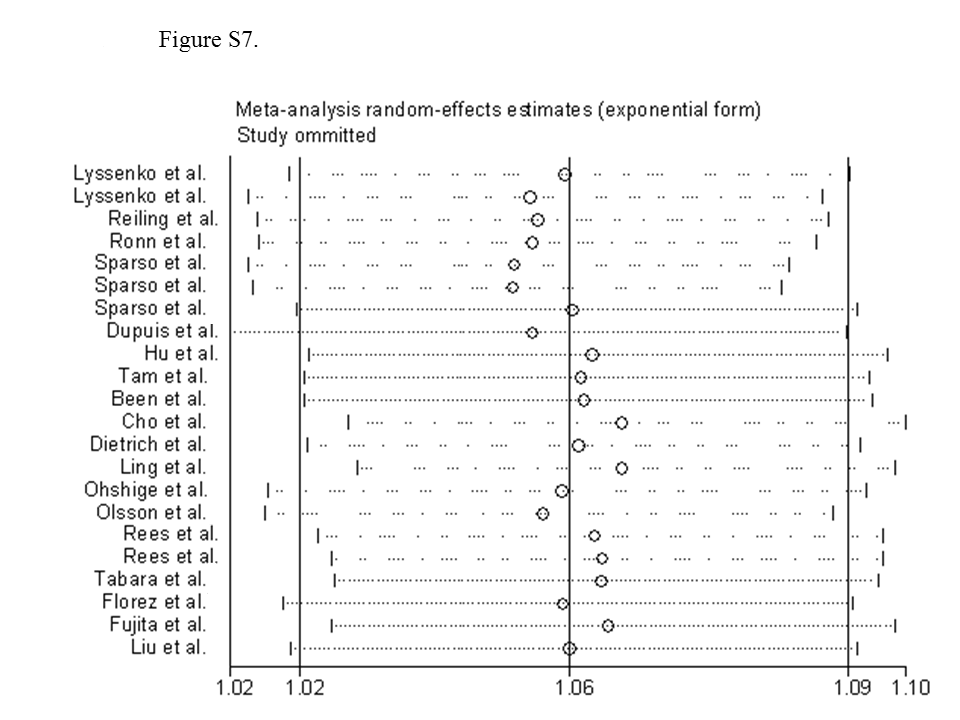

Supplement: Figure S7 — Sensitivity analyses of the MTNR1B rs10830963 variant in an additive model by omitting one study at a time. The summary OR (95% CI) was indicated by each horizontal line when the labeled study was omitted and the reminders were reanalyzed. (TIF) [file pone.0067665.s007.tif]

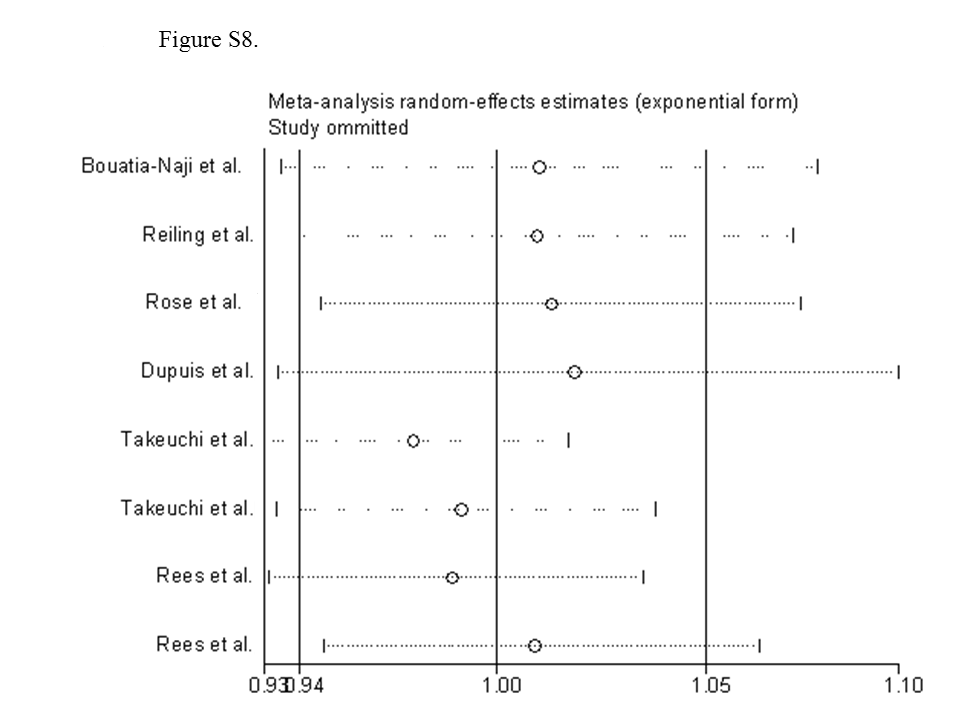

Supplement: Figure S8 — Sensitivity analyses of the G6PC2 variant in an additive model by omitting one study at a time. The summary OR (95% CI) was indicated by each horizontal line when the labeled study was omitted and the reminders were reanalyzed. (TIF) [file pone.0067665.s008.tif]
