# Supplementary material for: Large Scale Meta-Analyses of Fasting Plasma Glucose Raising Variants in GCK, GCKR, MTNR1B and G6PC2 and Their Impacts on Type 2 Diabetes Mellitus Risk
Source: PLoS One. 2013 Jun 28;8(6):e67665. doi: 10.1371/journal.pone.0067665 (PMC3695948; doi:10.1371/journal.pone.0067665)
Supplement: Table S1 — Quality score assessment criteria. (DOCX) [file pone.0067665.s009.docx]

| **Table S1. Quality score assessment criteria** | |
| --- | --- |
| **Cretia** | **Score** |
| **Representativeness of cases** | |
| Consecutive/randomly selected from case population with clearly deﬁned sampling frame | 2 |
| Consecutive/randomly selected from case population without clearly deﬁned sampling frame or with extensive inclusion/exclusion criteria | 1 |
| No method of selection described | 0 |
| **Representativeness of controls** | |
| Controls were consecutive/randomly drawn from the same sampling frame (ward/community) as cases | 2 |
| Controls were consecutive/randomly drawn from a different sampling frame as cases | 1 |
| Not described | 0 |
| **Ascertainment of type 2 diabetes** | |
| Diagnosis of T2D by OGTT test or by medical history/treatment | 2 |
| Diagnosis of T2D by FPG test or by medical history/treatment | 1 |
| Diagnosis of T2D by patient self-report | 0 |
| **Ascertainment of controls** | |
| Controls were OGTT-tested to screen out T2D | 2 |
| Controls were FPG-tested to screen out T2D | 1 |
| No objective testing for the ascertainment of controls or not described | 0 |
| **Hardy-Weinberg equilibrium** | |
| Hardy-Weinberg equilibrium in control group | 2 |
| Depart from Hardy-Weinberg equilibrium in control group | 1 |
| No checking for Hardy-Weinberg equilibrium | 0 |
| **Genetic association assessment** | |
| Genetic association was assessed by appropriate statistics with adjustment for confounders | 2 |
| Genetic association was assessed by appropriate statistics without adjustment for confounders | 1 |
| Inappropriate statistics used | 0 |
