# Supplementary material for: Large Scale Meta-Analyses of Fasting Plasma Glucose Raising Variants in GCK, GCKR, MTNR1B and G6PC2 and Their Impacts on Type 2 Diabetes Mellitus Risk
Source: PLoS One. 2013 Jun 28;8(6):e67665. doi: 10.1371/journal.pone.0067665 (PMC3695948; doi:10.1371/journal.pone.0067665)
Supplement: Table S2 — Estimation of the pooled prevalence of the risk A-allele of GCK rs1799884. (DOCX) [file pone.0067665.s010.docx]

| **Table S2. Estimation of the pooled prevalence of the risk A-allele of GCK**  **rs1799884** | | | |
| --- | --- | --- | --- |
| **Study** | **Ethnicity** | **A allele frequence** | **Total number** |
| **Caucasian** |  |  |  |
| Cauchi et al. | French+Swiss | 0.18 | 4472 |
| Dupuis et al. | European | 0.16 | 87022 |
| Holmkvist et al. | Finnish | 0.12 | 2293 |
| Holmkvist et al. | Swedish | 0.16 | 13666 |
| Reiling et al. | Netherlands | 0.17 | 2041 |
| Rose et al. | Danish | 0.16 | 4441 |
| Vaxillaire et al. | French | 0.18 | 2251 |
| Pooled prevalence | | 0.16 | 116186 |
| **Asian** |  |  |  |
| Qi et al. | Chinese | 0.23 | 1908 |
| Onuma et al. | Japanese | 0.16 | 402 |
| Tam et al. | Chinese | 0.18 | 1644 |
| Hu et al. | Chinese | 0.22 | 3412 |
| Rees et al. | South Asian | 0.15 | 417 |
| Rees et al. | South Asian | 0.16 | 1167 |
| Ohshige et al. | Japanese | 0.18 | 2125 |
| Tabassum et al. | Asian Indian | 0.11 | 4588 |
| Tabassum et al. | Indo-European | 0.13 | 1209 |
| Iwata et al. | Japanese | 0.18 | 859 |
| Fujita et al. | Japanese | 0.21 | 2050 |
| Pooled prevalence | | 0.16 | 19781 |
| **Others** |  |  |  |
| Ezzidi et al. | Tunisian | 0.20 | 513 |
| Ng et al. | African American | 0.10 | 4265 |
| Cauchi et al. | Moroccan | 0.18 | 1055 |
| Cauchi et al. | Tunisian | 0.20 | 942 |
| Pooled prevalence | | 0.12 | 6775 |
