# Supplementary material for: Large Scale Meta-Analyses of Fasting Plasma Glucose Raising Variants in GCK, GCKR, MTNR1B and G6PC2 and Their Impacts on Type 2 Diabetes Mellitus Risk
Source: PLoS One. 2013 Jun 28;8(6):e67665. doi: 10.1371/journal.pone.0067665 (PMC3695948; doi:10.1371/journal.pone.0067665)
Supplement: Table S3 — Estimation of the pooled prevalence of the risk C-allele of GCKR rs780094. (DOCX) [file pone.0067665.s011.docx]

| **ESM Table S3. Estimation of the pooled prevalence of the risk C-allele of**  **GCKR rs780094** | | | |
| --- | --- | --- | --- |
| **Study** | **Ethnicity** | **C allele frequence** | **Total number** |
| **Caucasian** |  |  |  |
| Sparso et al. | Danish | 0.65 | 4891 |
| Bi et al. | White American | 0.6 | 3188 |
| Mohas et al. | Hungarian | 0.53 | 172 |
| Dupuis et al. | European | 0.62 | 87022 |
| Pooled prevalence | | 0.62 | 95273 |
| **Asian** |  |  |  |
| Qi et al. | Chinese | 0.56 | 1908 |
| Hu et al. | Chinese | 0.54 | 3412 |
| Onuma et al. | Japanese | 0.59 | 402 |
| Tam et al. | Chinese | 0.54 | 1644 |
| Wen et al. | Chinese | 0.52 | 1136 |
| Ling et al. | Chinese | 0.57 | 1161 |
| Rees et al. | South Asian | 0.72 | 1167 |
| Rees et al. | South Asian | 0.74 | 417 |
| Iwata et al. | Japanese | 0.59 | 859 |
| Tabassum et al. | Indo-European | 0.77 | 1209 |
| Tabassum et al. | Asian Indian | 0.81 | 4588 |
| Pooled prevalence | | 0.67 | 17903 |
| **Others** |  |  |  |
| Bi et al. | Black American | 0.82 | 9937 |
| Ng et al. | African American | 0.82 | 4265 |
| Pooled prevalence | | 0.82 | 14202 |
