# Supplementary material for: Large Scale Meta-Analyses of Fasting Plasma Glucose Raising Variants in GCK, GCKR, MTNR1B and G6PC2 and Their Impacts on Type 2 Diabetes Mellitus Risk
Source: PLoS One. 2013 Jun 28;8(6):e67665. doi: 10.1371/journal.pone.0067665 (PMC3695948; doi:10.1371/journal.pone.0067665)
Supplement: Table S4 — Estimation of the pooled prevalence of the risk G-allele of MTNR1B rs10830963. (DOCX) [file pone.0067665.s012.docx]

| **Table S4. Estimation of the pooled prevalence of the risk G-allele of**  **MTNR1B rs10830963** | | | |
| --- | --- | --- | --- |
| **Study** | **Ethnicity** | **G allele frequence** | **Total number** |
| **Caucasian** |  |  |  |
| Dietrich et al. | German | 0.27 | 547 |
| Lyssenko et al. | Finnish | 0.28 | 2632 |
| Lyssenko et al. | Swedish | 0.29 | 13998 |
| Olsson et al. | Norwegian | 0.29 | 1447 |
| Reiling et al. | Dutch | 0.25 | 2041 |
| Sparso et al. | French | 0.27 | 2894 |
| Sparso et al. | French | 0.28 | 4343 |
| Sparso et al. | Danish | 0.36 | 4905 |
| Dupuis et al. | European | 0.30 | 87022 |
| Pooled prevalence | | 0.30 | 119829 |
| **Asian** |  |  |  |
| Ronn et al. | Chinese | 0.41 | 1105 |
| Hu et al. | Chinese | 0.43 | 3412 |
| Tam et al. | Chinese | 0.44 | 1644 |
| Been et al. | Asian Indian | 0.39 | 1021 |
| Ling et al. | Chinese | 0.40 | 1161 |
| Ohshige et al. | Japanese | 0.41 | 2125 |
| Rees et al. | South Asian | 0.42 | 1167 |
| Rees et al. | South Asian | 0.39 | 417 |
| Tabara et al. | Japanese | 0.41 | 402 |
| Fujita et al. | Japanese | 0.42 | 2050 |
| Liu et al. | Chinese | 0.42 | 2786 |
| Pooled prevalence | | 0.42 | 17290 |
