# Supplementary material for: Large Scale Meta-Analyses of Fasting Plasma Glucose Raising Variants in GCK, GCKR, MTNR1B and G6PC2 and Their Impacts on Type 2 Diabetes Mellitus Risk
Source: PLoS One. 2013 Jun 28;8(6):e67665. doi: 10.1371/journal.pone.0067665 (PMC3695948; doi:10.1371/journal.pone.0067665)
Supplement: Table S5 — Estimation of the pooled prevalence of the risk A-allele of G6PC2 rs560887. (DOCX) [file pone.0067665.s013.docx]

| **Table S5. Estimation of the pooled prevalence of the risk A-allele of**  **G6PC2 rs560887** | | | |
| --- | --- | --- | --- |
| **Author** | **Race** | **A allele frequency** | **Total number** |
| **Caucasian** | |  |  |
| Bouatia-Naji et al. | French | 0.30 | 4073 |
| Dupuis et al. | European | 0.30 | 87022 |
| Reiling et al. | Dutch | 0.31 | 2041 |
| Rose et al. | Danish | 0.31 | 4773 |
| Pooled prevalence | | 0.30 | 97909 |
| **Asian** |  |  |  |
| Rees et al. | South Asian | 0.16 | 417 |
| Rees et al. | South Asian | 0.18 | 1167 |
| Takeuchi et al. | Japanese | 0.03 | 6406 |
| Takeuchi et al. | Sri Lankan | 0.10 | 515 |
| Pooled prevalence | | 0.04 | 8505 |
