# Supplementary material for: Large Scale Meta-Analyses of Fasting Plasma Glucose Raising Variants in GCK, GCKR, MTNR1B and G6PC2 and Their Impacts on Type 2 Diabetes Mellitus Risk
Source: PLoS One. 2013 Jun 28;8(6):e67665. doi: 10.1371/journal.pone.0067665 (PMC3695948; doi:10.1371/journal.pone.0067665)
Supplement: Table S7 — PRISMA Flow Diagram for the current meta-analysis. (DOC) [file pone.0067665.s015.doc]

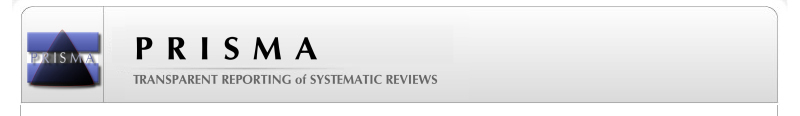
**PRISMA 2009 Flow Diagram**

**Screening**

**Included**

**Eligibility**

**Identification**

Records identified through database searching
(n =509)

Additional records identified through other sources
(n =0)

Records after duplicates removed
(n =509)

Records screened
(n =53)

Records excluded
(n =456)

Full-text articles assessed for eligibility
(n =53)

Full-text articles excluded, with reasons
(n =15)

Studies included in qualitative synthesis
(n =38)

Studies included in quantitative synthesis (meta-analysis)
(n =38)
